# First person – Jessica Sam

**DOI:** 10.1242/bio.054247

**Published:** 2020-06-24

## Abstract

First Person is a series of interviews with the first authors of a selection of papers published in Biology Open, helping early-career researchers promote themselves alongside their papers. Jessica Sam is first author on ‘Specificity, redundancy and dosage thresholds among gata4/5/6 genes during zebrafish cardiogenesis’, published in BiO. Jessica is a PhD student in the lab of Todd Evans at the Department of Surgery, Weill Cornell Medicine, New York, USA, investigating the genetic mechanisms underlying cardiac development and congenital heart disease.


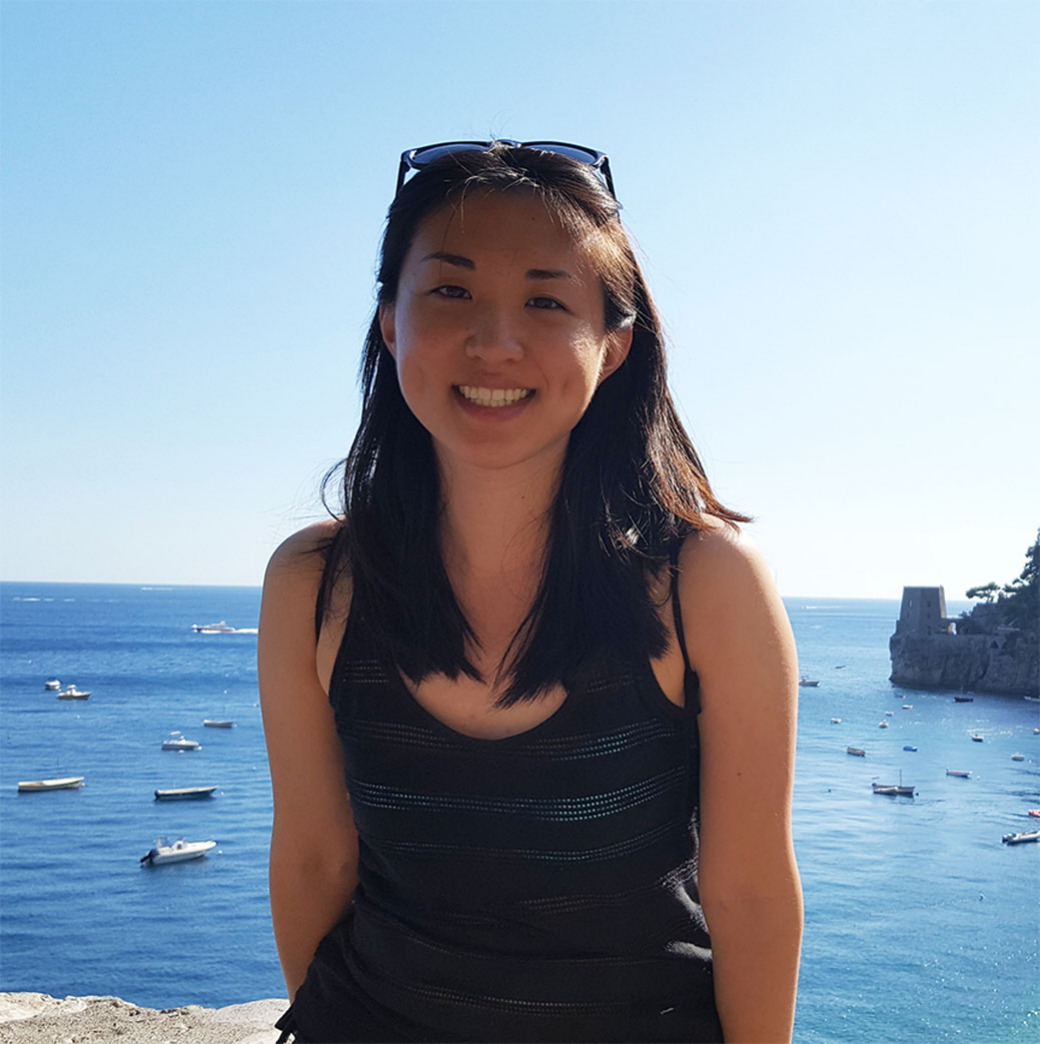


**Jessica Sam**

**What is your scientific background and the general focus of your lab?**

I am currently pursuing my PhD in Cell and Developmental Biology under the mentorship of Dr Todd Evans. In the Evans lab, we utilize a combination of embryonic stem cell models and zebrafish to investigate the underlying mechanisms that drive hematopoietic and cardiovascular development. Overall, we aim to elucidate how disruptions in the molecular pathways regulating organ morphogenesis can give rise to developmental defects and disease. My project in particular is focused on defining how individual and specific combinations of Gata4/5/6 transcription factors function to control early cardiogenesis in the fish. By examining how these genes contribute to building the heart, we can hopefully gain insight into how alterations in their expression cause cardiac abnormalities in humans.

**How would you explain the main findings of your paper to non-scientific family and friends?**

The heart is the first organ to form during embryogenesis and disruptions in its development can cause severe and sometimes lethal heart complications. The *GATA4*, *GATA5* and *GATA6* genes are essential for proper heart development and mutations in each are linked to a variety of congenital heart diseases in humans. However, how these mutations directly cause these diseases is poorly understood as all three genes have similar structures and expression patterns. In our study, we generated novel lines of *gata4/5/6* mutant zebrafish and characterized how different combinations of *gata* mutations disrupt each stage of early heart development. Specifically, we found that a progressive decrease in total *gata* expression leads to a severe loss of cardiac cells. We also found that loss of *gata6* expression alters the size of the ventricular chamber of the heart while the loss of *gata4* expression can cause age-dependent cardiomyopathy. Overall, our work provides new models that other scientists can use to study Gata function and our findings help shed light on how each of the three genes contributes to a specific aspect of heart formation.

**What are the potential implications of these results for your field of research?**

GATA4, GATA5 and GATA6 play key roles in generating mesoderm and endoderm derived organs. By creating targeted *gata4/5/6* deletion lines in the fish, we provide a new model for investigating Gata function in early embryogenesis. Similar mutations are often embryonic lethal in mice and up until now most studies in the fish have relied on morpholino knockdowns. Using our zebrafish lines, new studies can be done to more easily assess how single, double and even triple *gata* mutations impair different stages of embryonic development.

**What has surprised you the most while conducting your research?**

I was surprised by the amount of luck that can be involved in making new scientific discoveries. In our study, we found that our *gata4* mutant fish could potentially be utilized as a model for studying age-dependent cardiomyopathy. However, based on our initial experiments we failed to find a strong phenotype in the early embryonic stages despite the fact that *Gata4* mutations are embryonic lethal in mice. If we had not chosen to measure Gata4 protein levels using tissue samples from older adult fish, we would not have observed the cardiac abnormalities in our mutants or discovered the link between Gata4 and adult heart function.

“I was surprised by the amount of luck that can be involved in making new scientific discoveries.”

**What, in your opinion, are some of the greatest achievements in your field and how has this influenced your research?**

The development of precise gene editing technologies such as CRISPR/Cas9 has vastly expanded the toolkit available to scientific researchers. The ability to create specific targeted mutations in the genome combined with the imaging capabilities of the zebrafish system provides us with a powerful model for conducting cardiovascular research. Using CRISPR and TALEN technology to create our *gata* mutant alleles gave us the opportunity to make genetic analyses that we were previously unable to accomplish with knockdown techniques and it was vital to making the discoveries regarding *gata* gene dosage and compensation presented in our paper.

**Figure:**
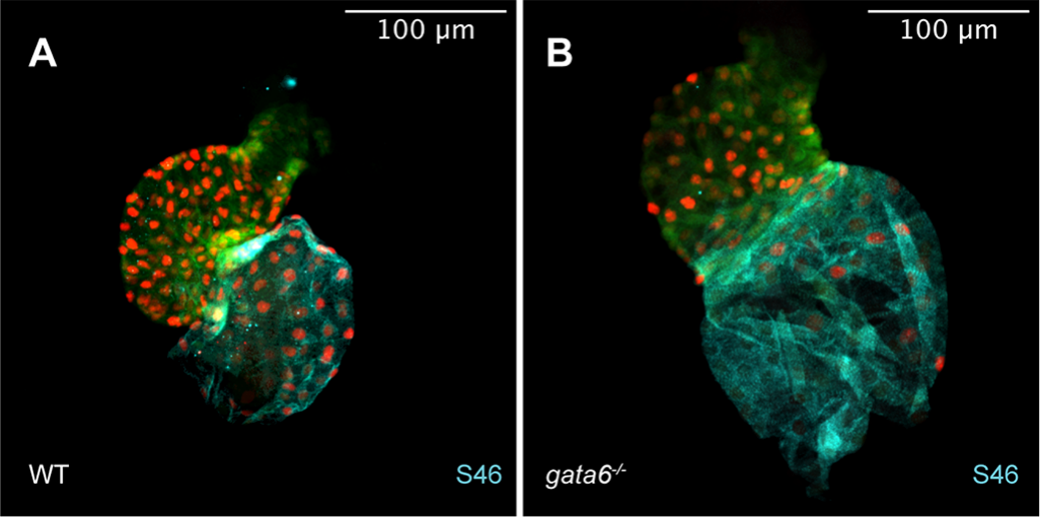
***Gata6* mutant embryos suffer defects in ventricular and atrial chamber morphogenesis.**

**What changes do you think could improve the professional lives of early-career scientists?**

Having more mentorship or networking opportunities with recent graduates working both in and outside of academia would be greatly beneficial. Building these connections would help younger scientists learn what to expect at each stage of an academic career and expose them to other science-related career possibilities as the pursuit of a nontraditional career track becomes increasingly more common. Developing these relationships would also help early-career scientists prepare for issues they will face both in and outside the lab setting, whether it is how to navigate the job market, handle the transition into post-doctoral life, or even just maintain a healthy work-life balance.

**What's next for you?**

In our paper, I identified exciting novel phenotypes for Gata6 and Gata4 in regulating embryonic and adult heart function, respectively. As I work towards completing my PhD studies, I hope to uncover the some of the underlying mechanisms involved in these phenotypes and better define the relationship between Gata factor function and the related cardiac defects found in human diseases.
